# Optical quantum confinement and photocatalytic properties in two-, one- and zero-dimensional nanostructures

**DOI:** 10.1098/rsos.180387

**Published:** 2018-09-12

**Authors:** T. Edvinsson

**Affiliations:** Department of Engineering Sciences, Solid State Physics, Uppsala University, Box 534, SE 751 21 Uppsala, Sweden

**Keywords:** quantum confinement, photocatalysis, quantum dots, hematite, ZnO

## Abstract

Low-dimensional nanomaterials have been explored extensively in the last decades, partly fuelled by the new possibilities for tuning and controlling their electronic properties. In a broader perspective within catalysis, two-, one- and zero-dimensional (2D, 1D and 0D) inorganic nanomaterials represent a bridge between the selectivity of molecular catalysts and the high performance and stability of inorganic catalysts. As a consequence of the low dimensions, higher surface areas are obtained but also introduce new physics and increased tuneability of the electronic states in the nanostructured system. Herein, we derive the commonly used equations for optical transitions and carrier confinement in semiconductors and discuss their effect on the optical and photocatalytic properties of direct band and indirect band gap materials. In particular, the physical properties of the optical and photocatalytic properties of Fe_2_O_3_ and ZnO will be used to exemplify the effects of the low dimensionality. Carrier confinement effects with changes in the density of states, band gap/shift of band edges will be outlined together with their effects on the tuneability of the material and their wider application as photocatalytic materials.

## 1. Introduction

Low-dimensional nanostructured materials are of interest as they provide a bridge between bulk and the molecular domain [1–3], providing entirely new avenues for controlling optical and electronic properties by size alone [1,4,5]. In a wider perspective, a material can be considered a nanostructured material if some of the dimensions are less than 500 nm, whereas larger domains normally are denoted sub-micron or micrometre sized. When a material exhibits a distinct change of electronic or optical properties as a result of a confinement in at least one dimension (1D), it is referred to as a quantum-confined structure. Here, one can distinguish these structures from the amount of confinement where two-dimensional (2D) materials, thin films and quantum wells (Q-wells), are confined in one dimension; 1D materials, quantum wires (Q-wires), are confined in 2D; and zero-dimensional (0D) materials, quantum dots (Q-dots), are confined in all three dimensions. A Q-dot is thus a 0D material and exhibits discrete quantized levels and different density of states compared to bulk and materials confined in fewer dimensions. At what size a material becomes quantum-confined can be quite different from material to material and is determined by the quantum mechanical nature of the electrons and holes in the materials. The quantum mechanical treatment of the optical processes and a formalism of how to quantify quantum confinement within the effective mass approximation will be given in §1.1 together with the effective density of states and other properties of the confined systems. Section 1.2 is devoted to the processes after the photoexcitation that are relevant for photocatalysis. In §2.1, we will briefly outline recent efforts in quantum confinement and photocatalysis in 2D hematite (α-Fe_2_O_3_) representing an indirect semiconductor, and in §2.2 we will outline optical quantum confinement and photocatalytic properties of 0D dimensional ZnO. In §3, the article ends with some summarizing conclusions and final remarks.

### 1.1. Optical transitions and density of states in low-dimensional semiconductors

To analyse the optical properties and the electronic transitions of low-dimensional materials in some detail, it is valuable to illuminate the different approximations involved in the conventional energy scaling equations for the direct and indirect transitions. This is important because it illuminates the different approximations applied in the commonly used expressions as well as identifying the main components affecting the band gap, absorption coefficient, and the density of states in low-dimensional systems. A quantum mechanical model of the absorption and creation of photogenerated charge carriers can be derived from the time-dependent polarization of the electron. The time-dependent Schrödinger equation for the interaction between light and an electron is given by Heitler [6]

1.1 $$[H_{0}(r)+H^{\prime }(t)]\psi =\left[\frac{p^{2}}{2m_{0}}+V(t)-\frac{e}{m_{0}}p\cdot A\right]\psi =i\hbar \frac{\partial \psi }{dt},$$

where *H*_0_ is the time-independent Hamiltonian, *ħ* is the reduced Planck's constant, *m*_0_ the electron mass, *e* the electron charge, ***p*** the electron momentum, ***V*** the potential, *t* the time, *r* the space coordinate and *ψ* the electron wave function. The factor –*e* · ***p*** · ***A***/*m*_0_ is the first-order time-dependent perturbation, *H*′(*t*), from the incoming oscillating electromagnetic field ***A***. The general solution to the time-dependent Schrödinger equation is

1.2 $$\psi (r,t)=\sum_{j}c_{j}(t)\psi (r)\exp\left(\frac{-iE_{j}t}{\hbar }\right),$$

for a system satisfying *H*_0_(***r***)*ψ*(***r***) = *E_j_ψ*(***r***) with the unperturbed Hamiltonian *H*_0_ = ***p***^2^/2*m*_0_ + ***V***(*t*), where *E_j_* is the energy of state *j* with the weighting factor *c_j_*. The coefficients *c_j_* can be obtained by solving the perturbation equation given in equation (1.1). Considering the electromagnetic field-electron interaction using a two-state model with the initial state, *i*, and the final state, *f*, the transition probability from the initial to the final state, *T*_i_*_→_*_f_, is given by the time derivative of the square of the weighting coefficient *c*_f_ [7]. Using the solution for *c*_f_ and using that for a long interaction time, the sine-squared function involved in the oscillating incoming field can be approximated by a delta function [8] and one can obtain the transition probability [9]

1.3 $$T_{i\to f}=\frac{\text{d}}{\text{d}t}|c_{f}(t){|}^{2}=\frac{2\pi }{\hbar }|H_{\text{if}}^{\prime }{|}^{2}\delta (E_{f}-E_{i}-\hbar \omega ),$$

which is the Fermi golden rule within the two-state approximation. Interestingly, the rule is named after Enrico Fermi although originally derived by Dirac [10]. In a semiconductor crystal, the electrons will experience a periodic potential which motivates the replacement of the ordinary wave function with Bloch functions whose eigenvalues are the band structure energies, *E_n_*(***k***), in reciprocal space and can also be extended to a perturbation approach [11]. If we consider the primitive cell in reciprocal space, the Brillouin zone (BZ), and the initial and final bands as the valence band energy, *E*_v_(***k***), conduction band edge, *E*_c_(***k***), and perform the same formalism for a two-state approximation within the band approximation in a crystal, equation (1.3) reformulates into

1.4 $$T_{v\to c}=\frac{\text{d}}{\text{d}t}|c_{f}(t){|}^{2}=\frac{2\pi }{\hbar }\int_{\mathrm{BZ}}\frac{1}{4\pi^{3}}|H_{\text{vc}}^{\prime }{|}^{2}\delta (E_{c}(k)-E_{v}(k)-\hbar \omega )\;\text{d}k,$$

where the integral is performed over the Brillouin zone with the normalization 1/4π^3^ coming from the 2/*Ω* number of spin states per unit volume 8π^3^/*Ω* of the primitive lattice cell. In the case of direct transition, there is no change in the crystal momentum between the initial and final states (*k*_f_*−k*_i_ = 0). The perturbation matrix element can then be considered independent of the *k*-vector within the first BZ when the wavelength of the perturbing light is much larger than the dimensions of the interaction volume in the BZ. *H′*_vc_ can then be placed outside the integration giving

1.5 $$T_{v\to c}=\frac{2\pi }{\hbar }|H_{\text{vc}}^{\prime }{|}^{2}\int_{\mathrm{BZ}}\frac{1}{4\pi^{3}}\delta (E_{c}-E_{v}-\hbar \omega )\;\text{d}k,$$

where the integral in equation (1.5) is

1.6 $$\int_{\mathrm{BZ}}\frac{1}{4\pi^{3}}\delta (E_{c}-E_{v}-\hbar \omega )\;\text{d}k=g_{\mathrm{vc}}(\hbar \omega ),$$

where *g*_vc_(*ħω*) is the joint density of states, which basically is the combination of donor states in the valence band and acceptor states in the conduction band. To be able to analyse this in the context of different materials and the changes upon quantum confinement, the energy levels within the delta function need to be specified. In a first approximation, the electrons and holes can, for energies close to the band edges, be described as particles obeying the energy of a harmonic potential, but with a modified or effective mass (m*) correcting for the changed ability to displace the charge carriers in the lattice. The valence and conduction band energies then obtain a parabolic dependence on the crystal momentum, ***k***, with *E*_c_(***k***) = *E*_c_(*0*) + *ħ*^2^***k***^2^/2*m*_e_* and *E*_v_(***k***) = *E*_v_(*0*) − *ħ*^2^***k***^2^/2m_h_* where *m*_e_* and *m*_h_* are the effective masses for the electrons and holes, respectively. As the effective masses for the charge carriers can be measured for different materials via the curvature of the bands from, e.g. the cyclotron resonance, angular resolved photoelectron spectroscopy or more indirectly via the conductivity that involves electron concentration and influence of possible defects, [12] this provides a link to the materials. Formally, the effective mass is a tensor and depends on the direction in the material but can, for an isotropic material without directional transport, be assumed to be a scalar. One can also obtain the effective mass from the band dispersion from quantum mechanical calculations via ${\left(m_{e}^{*}\right)}^{-1}=\hbar^{2}\partial^{2}E_{c,\;\min}(k)/\partial k^{2}$ and the corresponding expression for the effective mass of the hole. This is of special interest for predicting properties of new materials not yet available experimentally [13,14]. In the case of momentum preserving transition, the energy difference, *ħω*, between an initial state with energy *E*_i_ and a final state with energy *E*_f_, can, in the parabolic approximation for bands, be expressed as

1.7 $$h\omega =E_{f}(k)-E_{i}(k)=E_{c}-E_{v}+\frac{\hbar^{2}k^{2}}{2m_{n}^{*}}+\frac{\hbar^{2}k^{2}}{2m_{p}^{*}}=E_{g}+\frac{\hbar^{2}k^{2}}{2m_{\mathrm{red}}^{*}},$$

where $m_{\mathrm{red}}^{*}=m_{n}^{*}\cdot m_{p}^{*}/(m_{n}^{*}+m_{p}^{*})$ is the reduced effective mass, *ω* is the frequency of the light and *E*_g_ is the optical band gap. Expressing *k* = (2m*_red_/*ħ*^2^)^1/2^ (*ħω* −*E_g_*)^1/2^ from (1.7) and replacing the integration variable *dk* in equation (1.6) with *d*(*E*_c_ − *E*_v_) one obtains [15,16]

1.8 $$g_{\mathrm{vc}}(\hbar \omega )=\frac{1}{2\pi^{2}}{\left(\frac{2m_{\mathrm{red}}^{*}}{\hbar^{2}}\right)}^{3/2}{\left(\hbar \omega -E_{g}\right)}^{1/2}.$$

The transition probability can now be expressed by combining equations (1.5), (1.6) and (1.8) into

1.9 $$T_{v\to c}=\frac{2\pi }{\hbar }|H_{\text{vc}}^{\prime }{|}^{2}g_{\mathrm{cv}}(\hbar \omega )=|H_{\text{vc}}^{\prime }{|}^{2}\frac{1}{\hbar \pi }{\left(\frac{2m_{\mathrm{red}}^{*}}{\hbar^{2}}\right)}^{3/2}{\left(h\omega -E_{g}\right)}^{1/2}.$$

To analyse the time-dependent behaviour further, one needs to specify the functional form of the perturbation. First, we know that the distance of the electrons to the nuclei is on the order of a few Ångström and is much smaller than the wavelength of the light and one can thus to a very good approximation conclude that the spatial variation of the electromagnetic field over the dimensions of the atom is constant at a fixed time. The space-dependent electric field potential can then be expanded in multipole moments e^−i**k**^^**·**^^**r**^ ≈ 1 − *i**k · r*** + ··· and keep only the first term referred to as the electric dipole (ED) approximation with ***A***(*r*) = ***A·* e***^−i**k**^*^**·**^^***r***^ ≈ ***A***. The electric quadrupole (EQ) and magnetic dipole (MD) are of the same magnitude and smaller than the electric dipole by the dimensionless factor *e*^2^/4*πε*_0_*ħ* ≈ 1/137 (known as the fine-structure constant). For dipole-allowed transitions, $\langle i|H_{\mathrm{ED}}|f\rangle \neq 0$ and the contribution from EQ and MD can be neglected. For dipole-forbidden transitions where $\langle i|H_{\mathrm{ED}}|f\rangle =\;0$ and $\langle i|H_{\mathrm{EQ}}|f\rangle \neq 0$ or $\langle i|H_{\mathrm{MD}}|f\rangle \neq 0$, there can still be a finite probability for transitions but with much smaller probability and collectively referred to as dipole-forbidden transitions. Here, we will only analyse the electric dipole-allowed transition, but it can be replaced by the EQ or MD perturbation for results needed for these transitions. Using the dipole approximation, the perturbation element as defined in equation (1.1) and that ***p*** *=* *m_o_* d***r***/d*t*, we have

1.10 $$H_{\mathrm{vc}}^{\prime }=-\frac{\text{i}\omega A}{m_{0}}\cdot \int \psi_{v}^{*}er\psi_{c}{\text{d}}^{3}r=-\frac{1}{m_{0}}{\mu^{\prime }}_{\mathrm{vc}}\cdot E_{0},$$

which is the perturbation Hamiltonian within the dipole approximation for a two-state band model and the transition dipole moment of the transition, ***μ****′*_vc_, defined from the fundamental electron charge and the expectation value of the displacement. For a given direction of the polarization field, ***E*_0_**, and an effective mass of the electron in the valence band, *m*_v_*, one can express the transition probability in terms of the scalar dipole and the magnitude of the field and obtain

1.11 $$T_{v\to c}=\frac{E_{0}^{2}\mu_{\mathrm{vc}}^{2}}{\hbar \pi \;m_{v}^{*}}{\left(\frac{2m_{\mathrm{red}}^{*}}{\hbar^{2}}\right)}^{3/2}{\left(h\omega -E_{g}\right)}^{1/2}.$$

The absorption coefficient, *α*(*ħω*), is defined as the energy absorbed per unit time and unit volume divided by the incoming energy flux in a media and thus as *α*(*ħω*) = *T*_v→c_/(***S***/*ħω*), where ***S***/*ħω* is the normalized Poynting vector describing the incoming photon flux [16,17]. An expression for the absorption coefficient of direct and indirect bulk semiconductors were first stated in a paper by Hall *et al.* [18] and in a subsequent conference proceeding [19]. Considerations of the interaction situation can also be found in Fan *et al.* in the same proceeding [20] and also in a review article from the same year [21]. The expressions with some slightly different forms can also be found in Brooks *et al.* [22] and Moss [23]. The indirect transition involves a change in crystal momentum where the incoming light is coupled with a phonon for this transition to occur. This essentially represents a two-particle interaction and is thus a less likely event compared to the direct transition. It is also more complicated to describe as the interaction Hamiltonian *H′*_vc_ in equation (1.10) is no longer independent of ***k*** and thus cannot be taken outside the integral in equation (1.4). The initial and final states can, in this case, be described by

1.12 $$E_{v}=E_{\alpha k}+\hbar \omega_{\mathrm{light}}+\hbar \omega_{q}$$

and

1.13 $$E_{c}=E_{\beta k+q},$$

where *ħω*_light_ is the energy of an absorbed photon, *ħω_q_* is the energy of an absorbed phonon, and *α* and *β* refer to a different Bloch band. Including both phonon absorption (*E_α**k**_* + *ħω**_q_***) and emission (*E_α**k**_* *−* *ħω**_q_***), and integrating over the states, one obtains an extra contribution of (*ħω* *−* *E*_g_)^3/2^ for an allowed indirect transition [18] resulting in the transition probability scales with [18,19] (*ħω* *−* *E*_g_)^2^. A plot of the energy of the incoming photons, *ħω*_light_, versus the square root of the absorption coefficient would thus give the band gap of the indirect transition if extrapolated down to zero absorption. Within the assumptions made in the outlined derivations, a general schematic expression for the transition probability is generally given by

1.14 $$T_{v\to c}=C_{1}{\left(\hbar \omega -E_{g}\right)}^{\eta },$$

where *C*_1_ is a constant, *η* = 1/2 for an allowed direct transition and 2 for an allowed indirect transition. This also holds for forbidden direct and indirect transitions where *η* = 2/3 and 3, respectively [19,24,25].

For quantum-confined systems, several of the parameters, assumed to be constant in bulk semiconductors, are instead dependent on the dimensions of the material. The band gap, band edge positions and density of state change due to quantum confinement [1,26] have an effect on the transition probability. The general form of the density of states, *ρ*(*E*) = d*N*(*E*)/d*E*, in quantum-confined structures can be deduced from the effective mass approximation *E* = *ħ*^2^*k*^2^/2m* where *k* = (2m*E/*ħ*^2^)^1/2^ and using that d*N*/d*E* = d*N*/d*E* · d*k*/d*E* with d*k*/d*E* = (2m*/*ħ*^2^)^1/2^ · *E*^−1/2^/2 which gives the density of states for bulk as

1.15 $$\rho_{3D}(E)=\frac{1}{2\pi^{2}}{\left(\frac{2m*}{\hbar^{2}}\right)}^{3/2}E^{1/2}.$$

Note that this is only for the density of states within a band and under the assumption that the energy can be expressed as a parabolic function of crystal momentum (*k*). For a 1D confined system (a 2D material), the density of states is instead effected by the decreased number of freedoms for the charge carriers mainly from *N*_2D_ = 2*πk*^2^/(2*π*)^2^ formally only determined by *k*^2^ in contrast to the *k*^3^ dependence in 3D (*N*_3D_ = 2 · 4*πk*^3^/(3(2*π*)^3^). In all cases, the number of states per unit space is given by the spin-degeneracy factor (2 for allowing double occupancy in each state by different spins) multiplied by the space spanned by *k* in the different dimensions divided by the area occupied by each state. Using the expression for the number of charge carriers confined into 2D and performing the analogous approach as in the 3D case, one obtains

1.16 $$\rho_{2D}(E)=\frac{m^{*}}{\pi \hbar^{2}},$$

which is independent of the energy. At first glance, this seem unreasonable, but recalling that the expression of the density of state is derived within a band, the density of states *below a state n* can be written

**Figure 1.**
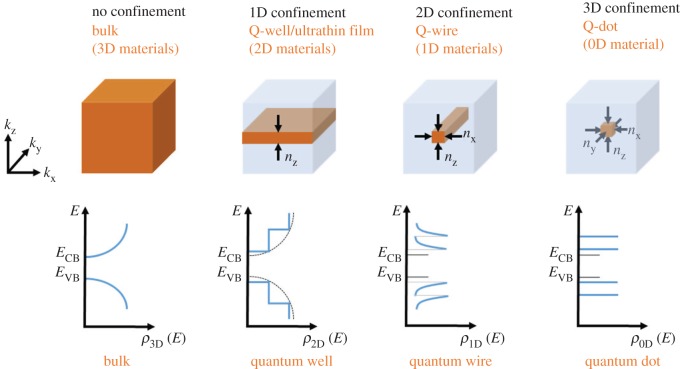
Schematic illustration of broken symmetry and functional form of the density of states in 1D, 2D and 3D confined materials.

1.17 $$\rho_{2D}(E)=\overset{n}{\underset{i=1}{\sum }}\frac{m^{*}}{\pi \hbar^{2}}H(E-E_{i}),$$

where *H* is the Heaviside step function which is zero for negative arguments and 1 for positive arguments. The density of states for the 1D and 0D materials can be derived analogously with *ρ*_1D_(*E*) = m*/*πħ* · (m*/2*E*)^−1/2^ and *ρ*_0D_(*E*) = 2*δE* with the latter formed only from the spin-degeneracy factor multiplied by the delta function of the energy levels in the energy range. Schematic illustrations showing the general form of the density of states for bulk, 2D, 1D and 0D materials are shown in figure 1.

The *effective* number of carriers at the bottom of a band, e.g. the conduction band (c) relevant for reduction reactions in photocatalysis can be formulated by integrating over the density of state multiplied by the probability of occupation with

1.18 $$N_{c}=\int_{E_{c}}^{E_{\infty }}\rho (E)f_{F}(E)\;\text{d}E.$$

where *f*_F_ is the Fermi–Dirac distribution function and can be replaced with the Boltzmann distribution function, *f*_B_, for room temperature situations and the elevated temperatures normally used in pollution control catalysis. Using 3D, 2D and 1D density of states in equation (1.15) and (1.16) and using that *f*_B_(*E*_c_) = *N*_c_e^−(*Ec*−*E*F)/*kT*^ where *E*_F_ is the Fermi level, one obtains

1.19 $$N_{c}^{3D}=\frac{1}{\sqrt{2}}{\left[\frac{m^{*}kT}{\pi \hbar^{2}}\right]}^{3/2},$$

1.20 $$N_{c}^{2D}=\frac{m^{*}kT}{\pi \hbar^{2}}$$

1.21 $$\;\text{and}\;N_{c}^{1D}=\sqrt{\frac{m^{*}kT}{2\pi \hbar^{2}}}.$$

where the delta function in *ρ*_1D_(*E*) = 2*δE* reduces $N_{c}^{0D}$ to 2 at the conduction band edge if occupied. An overview of the dimensionality of the materials, the dispersion relation and the corresponding density of states and effective density of states close to a conduction band minima is given in table 1. For an anisotropic band structure around the minima, the effective mass scalar has to be replaced with the effective mass tensor representing the correct dispersion in the different directions in *k*-space. For an oxidation reaction, the density of states of the holes in the valance band (v) is more relevant and is analogous with respect to quantum confinement to the density of states of the electrons but with the effective mass of the holes and integrating from *E*_v_ instead. Here, one can also take the effective mass of the light- (lh) and heavy hole (hh) into account via a summation of the states to obtain [27]

1.22 $$N_{v}^{3D}=\frac{1}{\sqrt{2}}{\left[\frac{m_{\mathrm{hh}}^{*}kT}{\pi \hbar^{2}}\right]}^{3/2}+\frac{1}{\sqrt{2}}{\left[\frac{m_{\mathrm{lh}}^{*}kT}{\pi \hbar^{2}}\right]}^{3/2},$$

and the corresponding summation for the 2D, 1D and 0D situation.

**Table 1.** The dimensionality of the materials as degrees of freedom, the dispersion relation and the corresponding density of states and effective density of states at the conduction band in low-dimensional semiconductors.

degrees of freedom	dispersion (kinetic energy)	density of states (close to the conduction band)	effective density of states (at the conduction band)
3D (bulk)	$E=\frac{{\bar{h}}^{2}}{2m^{*}}(k_{x}^{2}+k_{y}^{2}+k_{z}^{2})$	$\rho_{3D}=\frac{1}{2\pi^{2}}{\left(\frac{2m*}{\hbar^{2}}\right)}^{\frac{3}{2}}{\left(E-E_{c}\right)}^{1/2}$	$N_{c}^{3D}=\frac{1}{\sqrt{2}}{\left[\frac{m^{*}kT}{\pi {\bar{h}}^{2}}\right]}^{3/2}$
2D (film)	$E=\frac{{\bar{h}}^{2}}{2m^{*}}(k_{x}^{2}+k_{y}^{2})$	$\rho_{2D}=\overset{n}{\underset{i=1}{\sum }}\frac{m^{*}}{\pi {\bar{h}}^{2}}\text{H}(E-E_{c})$	$N_{c}^{2D}=\frac{m^{*}kT}{\pi {\bar{h}}^{2}}$
1D (wire)	$E=\frac{{\bar{h}}^{2}}{2m^{*}}(k_{x}^{2})$	$\rho_{1D}=\frac{1}{m^{*}\pi \bar{h}}{\left(\frac{m^{*}}{2(E-E_{c})}\right)}^{1/2}$	$N_{c}^{1D}=\sqrt{\frac{m^{*}kT}{2\pi {\bar{h}}^{2}}}$
0D (dot)	^a^	$\rho_{0D}=2\delta (E-E_{c})$	$N_{c}^{0D}=2$

^a^The dispersion is formally not defined in a 0D (3D confined) system as there is no periodicity in any direction.

**Figure 2.**
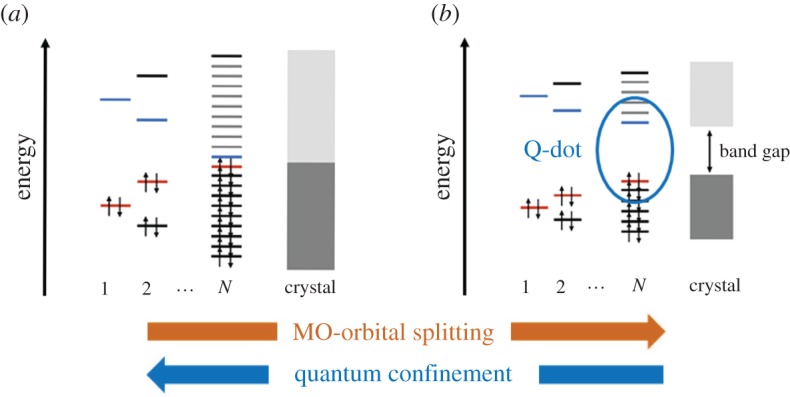
Schematic image of the quantum confinement effect in a metal (*a*) and a semiconductor (*b*).

One of the striking effects in quantum-confined materials is the size dependence of the electronic states and thus also the optical properties. When a metal particle decreases in size and eventually becomes a cluster, discrete states evolve [28–30]. This has remarkable effects on the infrared absorption for these structures and has been shown to require a quantum mechanical description that goes beyond a Drude model [31]. Sufficiently small metallic clusters more or less behave as semiconductors, as illustrated in figure 2. Conversely, low-dimensional materials that natively are crystalline semiconductors in bulk also lack sufficiently repetitive crystal lattice to converge into dense bands and a band gap energy corresponding to the bulk bandgap (figure 2*b*). This can be described by a bottom-up principle from molecular orbital theory and splitting of orbital energies in transformation from molecular orbitals to cluster orbitals, to eventually form dense bands for larger crystals. The phenomenon can also be described through the notion of quantum confinement of the bulk material in a top-down model. The latter has the advantage that it can be parametrized from known bulk properties such as the effective mass and dielectric constant of the material and also predicts when the electronic states of the materials will experience quantum confinement.

In a first approach to describing the optical properties in quantum-confined semiconductors by Brus [1], the photogenerated electron hole pair is approximated with a model Hamiltonian for a bound state within the effective mass approximation in an effective potential surrounding, given by [1,4]

1.23 $$\hat{H}=\frac{-\hbar^{2}}{2m_{e}^{*}}+\;\nabla_{e}^{2}\frac{-\hbar^{2}}{2m_{h}^{*}}\nabla_{h}^{2}-\frac{e^{2}}{\varepsilon |r_{e}-r_{h}|}+\;\overset{\infty }{\underset{n=1}{\sum }}\alpha_{n}\frac{e^{2}}{2}\frac{\left(r_{e}^{2n}+r_{h}^{2n}\right)}{R^{2n+1}},$$

where m*_e_ and m*_h_ are the effective masses for the electron and hole*, ɛ* is the dielectric constant, *α* the polarizability, *r*_e_ and *r*_h_ are the positions for the electron and the hole within a spherical potential well with radius *R,* representing the particle radius. Following Brus, we can assume that the wave functions for the electrons and holes can be separated as *Ψ* = *Φ*(*r*_e_)*Φ*(*r*_h_) and thus that the correlation between the electron and hole is small. The summation term in equation (1.23) is the effective polarization and can be considered angular independent in isotropic particles. Without the polarization term, equation (1.23) reduces to the screened Wannier Hamiltonian for a bound electron-hole pair. Solving the Schrödinger equation for the first exited state for an electron-hole pair confined within a radius *R* within the effective mass approximation for the kinetic energy and an effective medium approach with dielectric screening for the potential energy, one obtains

1.24 $$E_{g}=E_{g,\mathrm{bulk}}+\frac{\hbar^{2}\pi^{2}}{2R^{2}}\left(\frac{1}{m_{e}^{*}}+\frac{1}{m_{h}^{*}}\right)-\frac{1.8e^{2}}{\varepsilon R}+\;\frac{e^{2}}{R}\overset{\infty }{\underset{n=1}{\sum }}\alpha_{n}{\left(\frac{r_{e}+r_{h}}{R}\right)}^{2n},$$

where the band gap of the material in the quantum-confined situation is described as a modification of the bulk band gap, *E*_g,bulk_.

**Figure 3.**
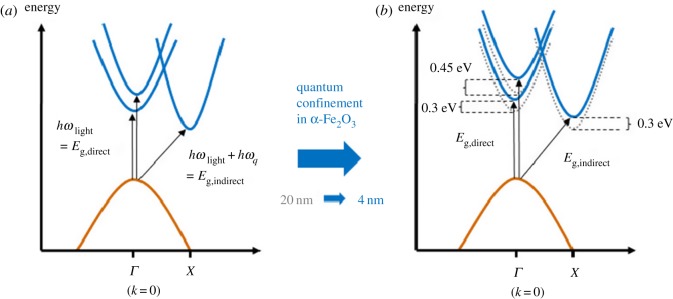
Reduced band dispersion diagram for α-Fe_2_O_3_ (*a*) and the corresponding shifts in bands seen when analysing the optical transitions in quantum-confined α-Fe_2_O_3_ (data adopted from Fondell *et al*. [9]).

The increased kinetic energy from the localization of the electron-hole pair inside a sphere with radius *R* is described in the second term and scales as *R*^−*2*^. The third term is the Coulomb attraction in a screened environment and scales as *R*^−1^. The polarization term, expressed as an average polarization, has the same scaling as the screened Coulomb attraction. This gives a functional dependence between the band gap, *E*_g_, and the particle diameter, *d*, given by *E*_g_ = *C*_1_ + *C*_2_/d + *C*_3_/*d*^2^, where the coefficients *C_n_* are to be found. The second constant (*C*_2_) comprises both the Coulomb and the polarization effect, whereas *C*_3_ gives the magnitude of band gap change from the confinement of the kinetic energy. Discussion of the parameters for ZnO quantum dots and different approaches to parametrize the band gap change in these systems are given in reference [4]. In an indirect semiconductor, such as α-Fe_2_O_3_ (hematite), the optical band gap onset is from an indirect transition depicted in figure 3. Deeper into the absorption profile, both direct and indirect transitions occur where the quantum confinement effect different bands differently, as illustrated in figure 3*b* where the indirect transition, in principle, disappears for ultrathin α-Fe_2_O_3_ films [9]. For an indirect semiconductor, the total optical quantum confinement is the combined effect of the electronic quantum confinement and the vibrational quantum confinement.

In general, using a hydrogenic wave function with the kinetic energy at the band minima described by an effective mass approximation and an effective medium surrounding for the potential, a material Bohr radius, *a*_B_, can be used as a measure of the average distance between the electron and holes in a material via [26]

1.25 $$a_{B}=\frac{E\hbar^{2}}{e^{2}}\left(\frac{1}{m_{e}^{*}}+\frac{1}{m_{h}^{*}}\right)=\frac{E}{m_{\mathrm{red}}^{*}/m_{0}}a_{0},$$

where *ɛ* is the dielectric constant of the material, *e* is the elementary charge, $m_{\mathrm{red}}^{*}$ is the reduced effective mass, *m*_0_ the mass of an electron and *a*_0_ the Bohr radius of hydrogen in Gaussian units. For a material system smaller than the Bohr radius *a*_B_ in at least 1D, the system can be said to be in the strong confinement regime, and if in comparable or larger dimensions, in the weak confinement regime [32].

### 1.2. Processes relevant for photocatalysis

Photocatalysis can basically be divided into four different processes. Light must be absorbed, charge carriers have to be separated, transported and have an energetically favourable chemical potential for the targeted reaction. The last factor is also dependent on a favourable pathway for the desired reaction at the semiconductor electrolyte interface (SEI), which implies a catalytically active site. The functions of the photocatalysis can then be specified as a product of these processes in an expression for the external quantum efficiency (EQE) via

1.26 $$\text{EQE}(\lambda )=\text{LHE}(\lambda )\cdot \Phi {\left(\lambda \right)}_{\mathrm{sep}}\cdot \Phi {\left(\lambda \right)}_{\mathrm{trans}}\cdot \Phi {\left(\lambda \right)}_{\mathrm{cat}},$$

where *λ* is the wavelength of light*,* LHE is the light harvesting efficiency, *Φ*_sep_ is the quantum efficiency for charge separation with respect to geminate recombination, *Φ*_trans_ is the quantum efficiency for carrier transport with respect to non-geminate recombination and *Φ*_cat_ is the efficiency of the catalytic processes at the surface. LHE(*λ*) is the absorption coefficient at wavelength *λ* times the thickness of the material and can be quite different for a quantum-confined material as the electronic states and the density of states is affected by the confinement. The quantum efficiency for charge separation and transport are in the most general case wavelength dependent (and indeed intensity dependent) as the separation of charges can occur in different electronic bands if excited at different wavelengths and also that the charges can be transported with different efficiency if generated further in the material or close to the surface. *Φ*_cat_ can be defined as the ratio of the total net flux of the photogenerated charges reaching the surface, *J*_surf_, and the charges reacting with the desired red-ox acceptor states in the solution, *J*_reac_ [33]. Ideally the absorber material is catalytic in itself, making *Φ*_cat_ = *J*_reac_/*J*_surf_ approach unity. Most probably, this is not the case and an additional co-catalyst is commonly deposited on the photoabsorber material. For a general electron transfer reaction, *J*_reac_ depends on the electron transfer rate constant, *k*_et_, which depends on the electronic coupling and the driving force according to Marcus, Sutin 1985, [34,35] as well as on the concentration of donor and acceptor states at the SEI, both of which are dependent on quantum confinement. The current reaching the surface, Jsurf, is also dependent on the dimensions and the material properties of the photocatalyst, and can be formulated as a summation of the diffusion (*j*_diff_) and migration currents (*j*_migr_) via

1.27 $$j_{\mathrm{surf}}=j_{\mathrm{diff}}+j_{\mathrm{migr}}=eI_{0}\left[\frac{\alpha L}{1+\alpha L}\right]{\text{e}}^{-\alpha d_{\mathrm{sc}}}+eI_{0}[1-{\text{e}}^{-\alpha d_{\mathrm{sc}}}]=eI_{0}\left[1-\frac{{\text{e}}^{-\alpha d_{\mathrm{sc}}}}{1+\alpha L}\right],$$

where *e* is the elementary charge, *I*_0_ the flux of the incident light, *α* the absorption coefficient, *d*_sc_ the thickness of the space-charge layer and *L* the average length of the minority charge carrier. This expression, together with consideration of the lifetime of the excess carrier, was first derived by Gärtner [36], assuming that the change in concentration of the charge carriers approaches zero when reaching the space-charge layer. Inclusion of the lifetime of the excess carrier gives a diffusion current complemented by –*en*_0_*D*/*L* where *n*_0_ is the equilibrium density of minority charge carriers, *D* *=* *L*^2^/*τ* is the diffusion coefficient for the minority carriers and *τ* is the lifetime of the excess carriers. Recalling the effective density of states in table 1, the effective diffusion and fields that can be generated for migration can be quite different for 3D, 2D, 1D and 0D materials. The diffusion current will decrease with increasing material thickness, while a successfully larger depletion (or accumulation) layer up to *d*_sc_ will be created. The directional dependence as well as the magnitude of this will be determined by both their dimensionality and the type of material used.

**Figure 4.**
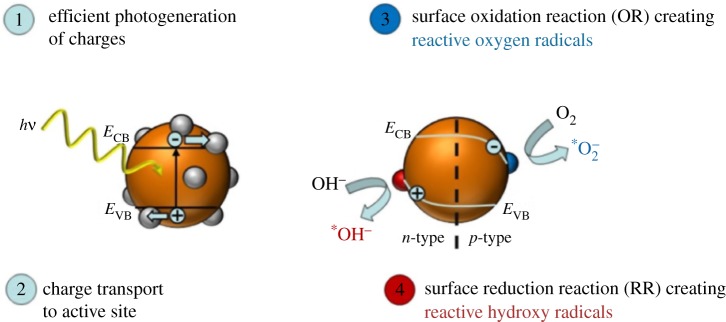
Schematic of the various processes in photocatalytic water splitting by a semiconductor particle with RR and OR catalysts on the surface and the involved energy levels.

The different processes in equation (1.26) are illustrated in figure 4 with the photogeneration of charges (1), the charge separation and transport merged in step (2), and the quantum efficiency of the interfacial charge transfer, which is divided into the oxidation reaction (OR) (3) and the reduction reaction (RR) (4).

A characteristic of wide band gap metal oxides such as TiO_2_ and ZnO is that the overpotential for the oxidizing potential of the holes is greater than the overpotential for the reduction to create reactive oxygen species (ROS). Starting with the Poisson equation and estimating the potential drop from the centre of a quantum dot with radius r to the surface, *ϕ*_r_, within the effective medium approximation, one has

1.28 $$\phi_{r}=\frac{eN_{D}}{6\varepsilon }r^{2}.$$

For a highly doped semiconductor, which also applies for unintentionally doped quantum particles, band bending can occur down to diameters of 3–4 nm for ZnO, [37] depending on doping density. If band bending is present, this will add local field facilitating a more effective charge transport (*Φ*_trans_) of the holes to the surface (recalling equation (1.27) and *j*_surf_ = *j*_diff_ + *j*_migr_) beneficial for oxidation reactions but detrimental for reduction reactions (figure 4*b*). In a water environment, hydroxyl groups adsorbed at the surface can be easily oxidized, therefore functioning as electron donors to the positively charged holes and forming hydroxyl radicals (*OH) which are extremely reactive. If the conduction band (CB) is high enough, radical anions are formed (*O_2_) (figure 4), which in e.g. photocatalytic water cleaning from organic pollutants, can also facilitate the formation of organic peroxy radicals in intermediate degradation compounds. The unstable product includes at least four oxide ions and can be broken down further, which enables the production of carbon dioxide and water as harmless end products. The radical anions increase the oxidation process and make it into a combustion process. A further mechanism is the formation of atomic oxygen, which is very reactive and attacks carbon–carbon bonds in organic compounds, degrading these materials. The reactive oxygen radicals can react with all types of organic substrates, such as aromatic and chlorinated compounds and microorganisms, and degrade them to intermediates such as aldehydes and carboxylic acids, which eventually are completely mineralized to the photocatalytic end products of carbon dioxide and water.

## 2. Quantum confinement and photocatalysis in indirect and direct semiconductors

### 2.1. Quantum confinement and photocatalysis in two-dimensional hematite

**Figure 5.**
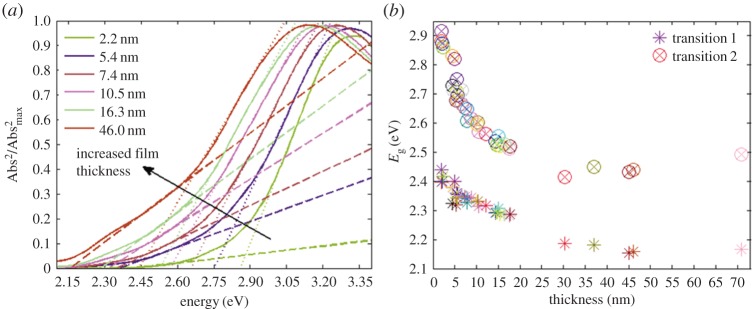
(*a*) Square of normalized absorption data against photon energy for ultrathin α-Fe_2_O_3_ films prepared with ALD. (*b*) Energy of the two direct transitions as a function of film thickness. Reproduced with permission from Fondell *et al*. [9] (Copyright © 2014 The Royal Society of Chemistry).

Hematite has been extensively studied in photocatalysis and we will not review the extensive literature here but instead refer the interested reader to other sources for this. We will instead provide some general results from optical quantum confinement and comment on how this will affect the possibilities and challenges in emerging photocatalytic applications. Nanostructured materials, in general, are extensively used in catalysts and photocatalysis. What is the added benefit of using quantum-confined structures? First, in the strongly confined regime, the indirect semiconductor material can become a direct semiconductor that has a stronger absorption coefficient at the optical absorption edge but also implies a shift of these states to higher energy as illustrated in figure 3*b* with the corresponding experimental data in figure 5. As the effective density of states is expected to be very sharp and not dependent on energy in a 2D material (table 1), this opens up for applications where one wants to target specific reactions that have a higher yield at certain overpotentials. Reduction of CO_2_ to formic acid (HCO_2_H) occurs at −0.85 V versus SCE and reduction to methanol (CH_3_OH) occurs at −0.62 V versus SCE, enabling, for example, targeting of specific reactions by using different sizes of the photocatalyst.

**Figure 6.**
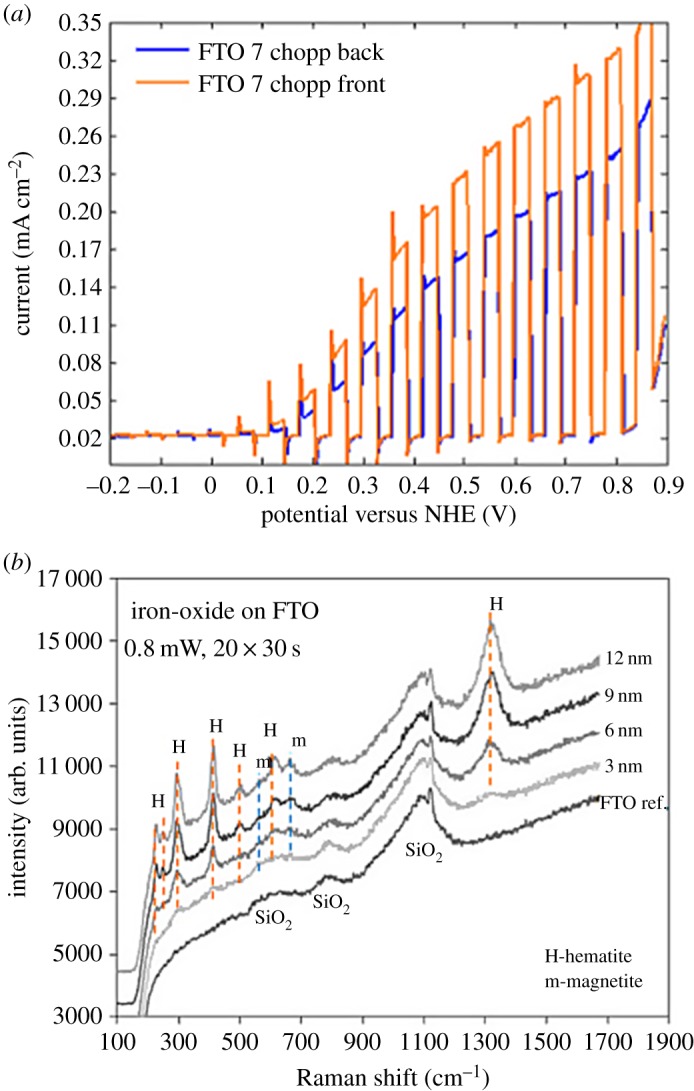
Resonance Raman spectra on ultrathin iron oxides prepared on FTO-glass (*a*). The spectra were recorded using the 514 nm line from an Ar-ion laser. The Raman spectra are Reproduced with permission from [40] (Copyright © 2015 American Chemical Society). (*b*) Photocatalytic water splitting with FTO/hematite electrodes using an AM1.5G solar simulator and 100 mWcm^−2^ intensity for backside (FTO/hematite) and frontside (hematite/FTO) illumination.

Hematite has been extensively used for photocatalytic water oxidation (using the photogenerated holes) but adding a bias voltage to promote the electrons to a higher energy to enable water reduction at a counter electrode band for the hydrogen evolution reaction (HER) [38,39]. If quantum confinement effects are used, less bias is needed but this also comes at the price of a larger bandgap where these effects need to be balanced to optimize the system. As for photocatalysts prepared for real applications, epitaxial growth cannot be used because the catalysts need to be very cheap in many applications. In practice, this implies that many photocatalysts are pseudo-2D, where quantum confinement is from the constraint of the crystals forming the 2D film [40]. Resonance Raman spectroscopy of ultrathin (hematite films 3–12 nm) made with atomic layer deposition (ALD) in our laboratory are shown in figure 6 together with photocatalytic water splitting on a thicker hematite film (60 nm) using backside (FTO/hematite) and frontside (hematite/FTO) illumination. The difference in photocatalytic current for frontside and backside illumination shows a limitation in transport properties (*Φ*_trans_ in equation (1.26)) in hematite when the charge carriers are produced far away from the surface. The intrinsic hole diffusion length in hematite has been known since the end of the 1970s to be very small (2–4 nm) [41] but depending on the type of electrolyte that is used, up to 20 nm depletion layer with band bending can occur in hematite. This renders an active thickness of a maximum 24 nm to effectively extract the charges to the surface with combined diffusion and drift. A small effect of the current decrease in figure 6 is also expected just from the different refractive index differences when going from electrolyte/FTO/hematite and electrolyte/hematite.

### 2.2. Quantum confinement and photocatalysis in 0D ZnO

**Figure 7.**
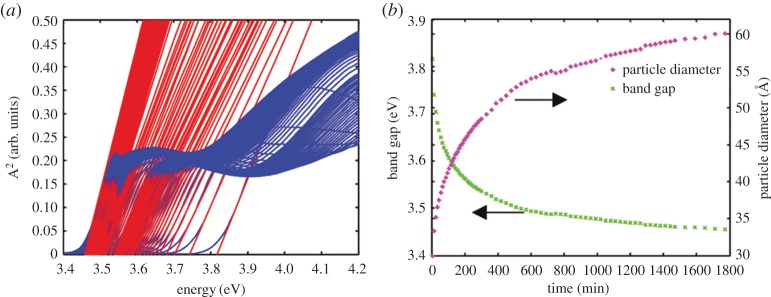
Change in bandgap for ZnO quantum dots prepared in solution (*a*) and bandgap particle size correlation (*b*). Reproduced with permission from [4] (Copyright © 2015 American Chemical Society).

Nanostructured ZnO is an attractive material that has applications spanning from UV absorbing additive in sunscreens and rubber, as a food additive, to more high-tech applications in chemical sensing, spintronics and photovoltaics. As with the case of hematite, we are not aiming to give a review of ZnO used for photocatalysis but instead aim to show a selection of properties that occur from quantum confinement that can be beneficial for photcatalysis. For comprehensive reviews on ZnO, in general, we refer to other sources [42–44]. The interest in ZnO for nano-scale optoelectronic applications is partly fuelled by the good carrier mobility, its direct wide band gap (*E*_g_ = 3.35 eV) and by the high exciton binding energy of 60 meV allowing room temperature exciton emission [43]. Also more intriguing aspects of quantum-confined ZnO, such as the extent of vibrational quantum confinement, [45] exciton surface stabilization, [46] and quantum-confined Stark effects [16] show that more is to be discovered even in seemingly well-known materials. Here, we will briefly outline how quantum confinement effects can be specifically used in emerging photocatalytic applications of ZnO. A recent study showed that formal quantum efficiency of photocatalysis is more effective in smaller ZnO quantum dots to an extent that goes beyond the increased surface area in these systems [47] and can be related to the interaction of excitons with charged surface states, [46] facilitating an improved possibility to break the exciton state. Figure 7 shows how the optical band gap changes during the growth of wurtzite ZnO quantum dots. The particles are shown with diameters from 30 Å (3 nm) to 60 Å (6 nm) where the band gap changes from 3.8 e to about 3.45 eV [4]. Bulk bandgap is achieved when the particles are around 9 nm in diameter (volume-averaged size).

**Figure 8.**
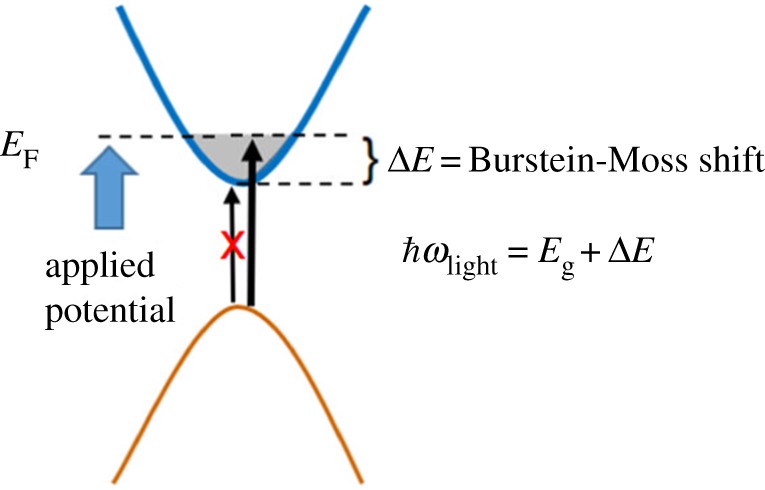
Illustration of a potentiostatically induced Burstein–Moss shift with an increased apparent optical bandgap as a result.

As the band gap changes, inevitably, the conduction band, valance band or both need to change as a result of the quantum confinement. Here, one runs in to the problem of how to determine the valence or conduction band edge with accuracy in situations corresponding to the photocatalysts in solution. Conventional techniques relying on X-ray photoelectron spectroscopy (XPS) valence band spectroscopy and X-ray absorption spectroscopy (XAS) require synchrotron facilities to go below 0.1 eV in resolution. As they are also intrinsically high vacuum techniques and have problem to measure with electrolytes (apart from recent XPS near-ambient pressure approaches allowing a monolayer of water) where the energy shifts can be significant (59 meV pH^−1^ unit), they can in some cases not be compared with the real condition of the photocatalysts under operation. The Mott–Schottky approach is also problematic because there is no flat surface and, for the smallest particles, no band bending. A promising approach is instead to use spectro-electrochemistry where state population induces a shift of the apparent optical bandgap. Identifying the potential where the shift starts, one can extract the energetic position of the conduction band edge as illustrated in figure 8.

**Figure 9.**
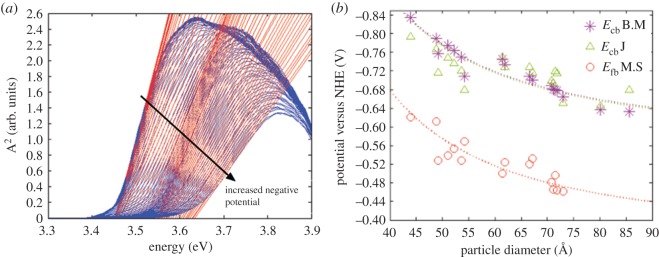
Potentiostatically induced Burstein–Moss shift in an electrode with a fixed size of the ZnO q-dots (*a*) and a master curve showing the conduction band energy versus NHE for ZnO quantum dots between 4 nm and 9 nm. B.M, J and M.S. denotes Burstein–Moss, Jacobsson *et al.* and Mott–Schottky in the figure captions. Reproduced with permission from [7] (Copyright © 2012 American Chemical Society).

Using this approach for a quantum dot electrode that has charge compensating ions in the solution that do not contribute to charge recombination, one obtains a shift in the apparent band gap induced by the applied potential as shown for potential dependent absorption spectroscopy in figure 9.

The analysis shows that the main effect for the quantum confinement occurs from a shift of the energy states in the conduction band [5] which then could be used to target different reactions or make an electrode with varying thresholds for charge injection in solar cell devices. Although the optical properties show a monotonic change with particle size for quantum-confined ZnO, the exciton emission shows a strong discontinuity at around 5 nm size [46] which seems to correlate with the growth changing from kinetically controlled along the *c*-axis to thermodynamically controlled, with more isotropic growth for particles larger than 4.5 nm [4]. The fluorescing trap levels are decoupled from the conduction band edge for particles smaller than about 4 nm, [4] while they are more or less at a constant energetic distance (about 0.32 eV) under the conduction band edge for quantum dots with sizes larger than about 5 nm (table 2) [48].

**Table 2.** Diameter and band gaps of ZnO quantum dots together with determination of the position of traps via potential dependent fluorescence spectroscopy [48].

diameter (nm)	*E*_g_ (eV)	peak position (V versus NHE)	distance of traps from *E*_c_ (eV)
4.8	3.53	−0.63	0.36
5.2	3.50	−0.55	0.41
5.7	3.47	−0.61	0.32
6.6	3.44	−0.57	0.32
7.5	3.41	−0.54	0.33
7.8	3.40	−0.54	0.32

**Figure 10.**
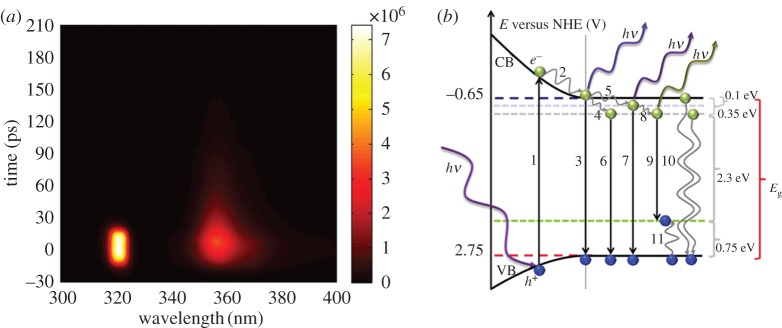
Streak camera response of ZnO Q-dots after a 320 nm pulse (*a*) and a schematic picture of the different processes that can occur. Reproduced with permission from [46] (Copyright © 2014 The Royal Society of Chemistry).

A streak camera image of the exciton emission pulsed by a 320 nm laser and a schematic picture of the processes are shown in figure 10 where the positions of the measured trap states are also included [46,48].

**Figure 11.**
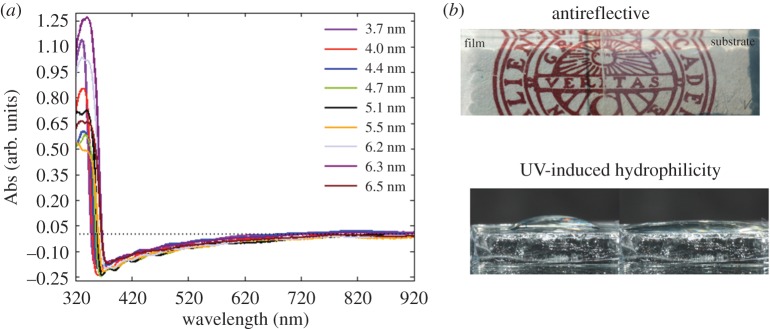
Antireflective behaviour for thin films of ZnO Q-dots with varying size (*a*) and photos of antireflective films and the hydrophilicity induced after 30 min exposure to UV-light (right). Reproduced with permission from [47] (Copyright © 2012 The Royal Society of Chemistry).

Using the combined thin film and quantum confinement properties, Q-dot films of approximately 100 nm thickness can be produced to give multifunctional material that is both antireflective and forms a hydrophilic surface after illumination (figure 11) beneficial for self-cleaning of glass or other photocatalytic applications.

**Figure 12.**
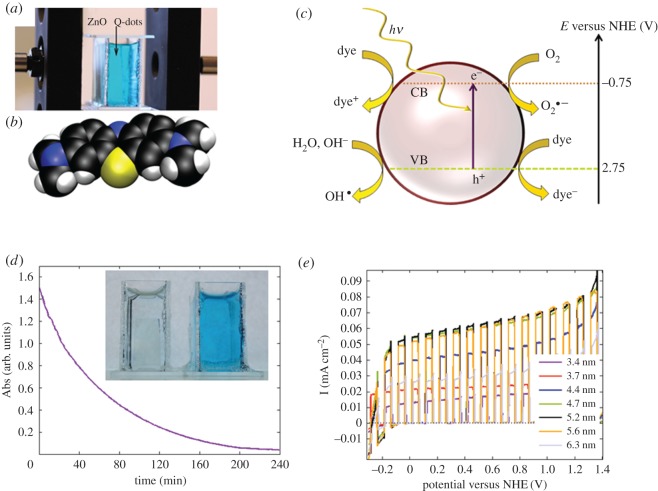
(*a*) A photo of a set-up for a photodecomposition experiment using ZnO Q-dot electrodes, (*b*) methylene blue, (*c*) illustration of the photodecomposition of an organic dye on a semiconductor nanoparticle and creation of ROS that further react with the dye in a water environment. (*d*) Monitoring the photocatalytic decolouration of a methylene blue water solution during 240 min and (*e*) Photocatalytic water splitting using ZnO quantum dots of different sizes. Reproduced with permission from [47] (Copyright © 2012 The Royal Society of Chemistry).

Using a material with a band gap in the UV with quantum confinement implies different shades of invisibility but also a control of the density of states and position of the band edges without doping or changing material composition. For artificial light sources such as UV-LEDs, the system is promising as a photocatalyst or if specific overpotentials are required to target specific reactions. For natural sunlight, on the other hand, the high band gap results in quite a few photons being absorbed in the solar spectrum although the formal quantum efficiency is higher for smaller particles,. This results in a trade-off at around 4.5 nm where the reactivity is still high, but the bandgap is lowered so that there are sufficient photons in the solar spectrum absorbed to photogenerate charges that can be used in the photocatalysis [47]. A picture, showing the set-up when performing water cleaning and photocatalytic water splitting with immobilized ZnO Q-dots on electrodes, is shown in figure 12.

Apart from heterogeneous photocatalysis in the liquid phase, the quantum-confined systems can also be used for the emerging field of photo-degradation of volatile organic compounds or as materials in photoactive sensors.

## 3. Conclusion and final remarks

Quantum-confined nanomaterials have been explored extensively in the last decade, to a large extent, motivated by the new possibilities for tuning and controlling the electronic properties. As the field contains equations and considerations from both solid-state theory and consideration of quantum confinement for the effective density of states to more practical situations, where light absorption, charge transfer and catalytic pathways for a reaction in solution or a gas phase are involved, we have here outlined the approximations involved in deriving the equations used in the field. The benefits of this are twofold. First, as different approximations are used in deriving the expressions commonly used in the field, the assumptions behind these approximations can be illuminated and give a critical view of the foundations of the equations and when they can be applied or need to be derived with new assumptions. In most cases, a two-state approximation would be valid if one considers the band edge properties, but in many applications there are light excitations occurring simultaneously from UV into IR where several band transitions and processes occur at the same time in the material. In these cases, or if one has phase mixtures, extensions to four-state models can be motivated [49]. As such, the contribution can be seen as a source for the expressions commonly used in optical quantum confinement without sacrificing the rigour in outlining the governing equations. Second, as the details of quantum confinement are seldom presented in the same sources as the processes used in photocatalysis, the article provides a convenient source for a perspective on the combination of the fields with the hope of spreading the use of these effects in emerging photocatalytic applications, bearing the promise of higher selectivity and targeting specific reactions. On the more practical side of this, results are presented for an indirect (α-Fe_2_O_3_) and direct semiconductor (ZnO) representing a 2D material (1D confined) and 0D material (3D confined) with some comments on how the quantum confinement can be used for emerging photocatalytic applications. For the use of 1D materials (2D confined) with respect to optical quantum confinement, there are very few examples in the literature, due to the challenge of producing these small dimensions in inorganic wires. The field of photocatalysis has a plethora of studies that refer to 1D dimensionality but then in context to the general dimension of a rod or wire, but not generally with sufficiently small dimensions to have 1D optically quantum-confined materials [50–53]. For organic matter, carbon nanotubes (CNTs) can partly be considered 1D but are intrinsically rolled-up 2D materials where the bandgap can be determined by the extension of the native 2D sheet, neglecting the strain in the 2D sheet from the curvature when rolled up into a tube [54,55]. In this context, carbyne (linear acetylenic carbon) is an intrinsic 1D system but shows small possibility of band gap control relevant for photocatalysis where instead bending is a more promising route towards optical change [56]. In a broader perspective within catalysis in general, two-, one- and 0D optically quantum-confined inorganic nanomaterials can find a place not only in approaches with increased control and selectivity but also in defect-driven catalysis, electrocatalysis or electrochemically promoted catalysts where the Fermi energy is changed via an external bias and can be optimized with a wider range of potentials than the ones obtained in a non-confined system.

## Supplementary Material

SI_Optical_Quantum_Confinement_Edvinsson.pdf

## Data accessibility

Data not included or fully reproduced in this article are available in the electronic supplementary material.
