# Supplementary material for: Optical quantum confinement and photocatalytic properties in two-, one- and zero-dimensional nanostructures
Source: R Soc Open Sci. 2018 Sep 12;5(9):180387. doi: 10.1098/rsos.180387 (PMC6170533; doi:10.1098/rsos.180387)
Supplement: SI_Optical_Quantum_Confinement_Edvinsson.pdf [file rsos180387supp1.pdf]

Supplementary information

Optical Quantum Confinement and Photocatalytic Properties in Two-, One-, and Zero-Dimensional Nanostructures

Tomas Edvinsson
Department of Engineering Sciences, Solid State Physics, Ångström Laboratory, Uppsala University, Box 534, 75121 Uppsala, Sweden

1. Key data for potential dependent absorption spectroscopy

Table S1. Key data for potential dependent absorption spectroscopy for ZnO quantum dots with diameters between 4.4 and 8.6 nm utilized for analysis of Burstein-Moss bleach discussed in main article. The potentials (V) are all reported *versus* the normal hydrogen electrode (NHE). The flatband potential (E_{fb}) reported in the table is determined by electrochemical impedance spectroscopy (EIS).

Sample	E_g [eV]	d [nm]	E_{cb} B (V)	E_{cb} J (V)	E_{fb} M 1225 Hz (V	E_{fb} M 3160 Hz (V)	dE_g/dU	U at I_{min} (V)
1	3.57	4.39	-0.83	-0.79	-0.60	-0.62	-1.30	-0.76
2	3.53	4.89	-0.79	-0.77	-0.59	-0.61	-1.16	-0.68
3	3.52	4.91	-0.76	-0.72	-0.50	-0.53	-1.30	-0.69
4	3.51	5.10	-0.77	-0.75	-0.53	-0.54	-1.48	-0.72
5	3.50	5.22	-0.76	-0.74	-0.54	-0.55	-1.46	-0.70
6	3.49	5.36	-0.75	-0.72	-0.51	-0.53	-1.15	-0.68
7	3.49	5.42	-0.71	-0.68	-0.50	-0.57	-1.26	-0.67
8	3.45	6.15	-0.75	-0.75	-0.49	-0.50	-0.68	-0.69
9	3.45	6.19	-0.73	-0.73	-0.51	-0.52	-0.76	-0.69
10	3.43	6.65	-0.71	-0.73	-0.49	-0.52	-0.57	-0.69
11	3.43	6.72	-0.70	-0.72	-0.52	-0.53	-0.72	-0.70
12	3.42	7.08	-0.69	-0.70	-0.46	-0.48	-0.74	-0.65
13	3.42	7.12	-0.68	-0.69	-0.45	-0.47	-0.85	-0.66
14	3.42	7.15	-0.68	-0.72	-0.49	-0.50	-0.50	-0.67
15	3.42	7.19	-0.68	-0.72	-0.45	-0.46	-0.60	-0.66
16	3.41	7.30	-0.66	-0.65	-0.44	-0.46	-0.52	-0.60
17	3.40	8.01	-0.64	-0.65	-0.67	-0.83	-0.84	-0.38
18	3.39	8.56	-0.63	-0.68	-0.86	-1.20	-0.48	-0.41

2. Derivation of the joint density of states, equation (8) in the main manuscript

Equation (4) in the main article is

$$T_{v\to c} = \frac{2\pi}{\hbar}\int_{BZ}\frac{1}{4\pi^3}\left|H'_{vc}\right|^2\delta\left(E_c(\boldsymbol{k})-E_v(\boldsymbol{k})-\hbar\omega\right)d\boldsymbol{k} \qquad \text{(S1)}$$

Considering a direct transition (no change in the crystal momentum between the initial, $\boldsymbol{k}_i$, and final states, $\boldsymbol{k}_f$), the perturbation matrix element between the corresponding states is then effectively independent of the *k*-vector within the BZ, and can be taken outside the integral to obtain

$$T_{v\to c} = \frac{2\pi}{\hbar}\left|H'_{vc}\right|^2 g_{vc}(\hbar\omega) \tag{S2}$$

where

$$g_{vc}(\hbar\omega) = \int_{BZ} \frac{1}{4\pi^3}\delta(E_c - E_v - \hbar\omega)d\boldsymbol{k} \tag{S3}$$

$g_{vc}(\hbar\omega)$ is here the joint density of states. Evaluation of the integral in (S3) can be performed by using the integral properties of the delta function

$$\int_a^b g(x)\delta(f(x))dx = \sum_{x_0} g(x_0)\left|\frac{\partial f(x)}{\partial x}\right|_{x=x_0} \tag{S4}$$

with $f(x_0)=0$ and $a<x_0<b$. To ensure that $f(x_0)=0$ in the interval [a,b]. A constant energy different surface between S and S+dS (between the valence and conduction band), valid at each k-point in the Brillouin zone have to be contructed with $d\boldsymbol{k}=dSdk_n$ where dkn is a wave vector normal to S. Since $|\nabla_k E|=dE/dk_n$, the constant energy difference can be expressed as

$$d\boldsymbol{k} = dSdk_n = dS\left[\frac{d(E_c - E_v)}{\left|\nabla_k (E_c - E_v)\right|_{Ec-Ev=\hbar\omega}}\right] \tag{S5}$$

Utilizing $d\boldsymbol{k}$ in (S3),(S4) and integrate over the constant $d(E_c-E_v)$ one obtain

$$g_{vc}(\hbar\omega) = \frac{1}{4\pi^3}\int \frac{dS}{\left|\nabla_k (E_c - E_v)\right|_{Ec-Ev=\hbar\omega}} \tag{S6}$$

and can be calculated if the functional form of E_c and E_v is known. Close to the band edges, the band structure energy can be expanded (with a truncation after the second order term) as

$$E_v(k) = E_v(k_0 + \kappa) = E_v(k_0) + \frac{1}{2}\sum_{\alpha\beta}\frac{\partial^2 E}{\partial k_\alpha \partial k_\beta}\kappa_\alpha\kappa_\beta \tag{S7}$$

$$E_c(k) = E_c(k_0 + \kappa) = E_c(k_0) + \frac{1}{2}\sum_{\alpha\beta}\frac{\partial^2 E}{\partial k_\alpha \partial k_\beta}\kappa_\alpha\kappa_\beta \tag{S8}$$

where k is the crystal momentum, k_0 is the crystal momentum at the band edge at the Γ-point E_v is the valence band energy, E_c is the conduction band energy, and κ is the crystal momentum in reciprocal space for two linear independent directions, a and β.

For isotropic parabolic dispersion close to the band edges, the electrons and holes can be described by a constant direction-independent parameter the effective mass), where the electrons and holes are described as effectively free carriers close band edges *via*

$$E_v(k) = E_v(0) - \frac{\hbar^2 k^2}{2m_p^*} \tag{S9}$$

$$E_c(k) = E_c(0) + \frac{\hbar^2 k^2}{2m_n^*} \qquad \text{(S10)}$$

with notations taken from the main article. Utilizing equation (7) in the main article and the energy difference in (S6) we have

$$\left|\nabla_k (E_c - E_v)\right| = \frac{\hbar^2 k}{m_{red}^*} \qquad \text{(S11)}$$

which together with (S6) gives the joint density of states

$$g_{vc}(\hbar\omega) = \frac{1}{4\pi^3}\left[4\pi k^2 \left(\frac{m_{red}^*}{\hbar^2 k}\right)\right]_{Ec-Ev=\hbar\omega} = \frac{1}{2\pi^2}\left(\frac{2m_{red}^*}{\hbar^2} k\right)_{Ec-Ev=\hbar\omega} \qquad \text{(S12)}$$

Expressing $k=(2m^*_{red}/\hbar^2)^{1/2}\ (\hbar\omega\text{-}E_g)^{1/2}$ we then have

$$g_{vc}(\hbar\omega) = \frac{1}{2\pi^2}\left(\frac{2m_{red}^*}{\hbar^2}\right)^{3/2} (\hbar\omega - E_g)^{1/2} \qquad \text{(S13)}$$

Which is equation (8) in the main article.

Justification:

A justification of eqn (S13) can be done by reformulating the density of states on the energy scale, $g(E)$, from the definition $g(E)dE=2g(k)dk$ where a factor of 2 is introduced from the two electron spins allowed for each k state. This gives $g(E)=2g(k)\ /\ (dE/dk)$ where dE/dk is the E-k dispersion curve valid under isotropic bands. The number of states per unit volume in k-space is $1/2\pi^3$ for the incremental volume between k and $k+dk$ $(4\pi k^2 dk)$ and give $g(k)=k^2/2\pi^2$.

In the parabolic approximation for the conduction band (given by eqn. (S8)) we have

$$g_c(E) = \frac{1}{2\pi^2}\left(\frac{2m_n^*}{\hbar^2}\right)^{3/2} (E - E_c)^{1/2} \qquad \text{(S14)}$$

The combined density of states, g_{if}, can then be introduce *ad hoc* within the parabolic approximation by combining equation (S12) with (S9) and (S10), which leads directly to the joint density of states expressed from the reduced effective mass and the bandgap

$$g_{vc}(h\omega) = \frac{1}{2\pi^2}\left(\frac{2m_{red}^*}{\hbar^2}\right)^{3/2} (\hbar\omega - E_g)^{1/2} \qquad \text{(S15)}$$

Although this leads to the same equation as equation (S13), the *ad hoc* replacement of separate effective masses to the reduced mass use the hidden assumption of the existence of a constant energy difference surface between the respective bands as shown above.
